# Parenteral Nutrition Combined with Enteral Nutrition for Severe Acute Pancreatitis

**DOI:** 10.5402/2012/791383

**Published:** 2012-12-11

**Authors:** Akanand Singh, Ming Chen, Tao Li, Xiao-Li Yang, Jin-Zheng Li, Jian-Ping Gong

**Affiliations:** ^1^Chongqing Key Laboratory of Hepatobiliary Surgery and Department of Hepatobiliary Surgery, The Second Affiliated Hospital of Chongqing Medical University, Chongqing 400010, China; ^2^Department of General Surgery, People's Hospital of Tongliang County, Tongliang, Chongqing 402560, China

## Abstract

*Background and Aims*. Nutritional support in severe acute pancreatitis (SAP) is controversial concerning the merits of enteral or parenteral nutrition in the management of patients with severe acute pancreatitis. Here, we assess the therapeutic efficacy of gradually combined treatment of parenteral nutrition (PN) with enteral nutrition (EN) for SAP. *Methods*. The clinical data of 130 cases of SAP were analyzed retrospectively. Of them, 59 cases were treated by general method of nutritional support (Group I) and the other 71 cases were treated by PN gradually combined with EN (Group II). *Results*. The APACHE II score and the level of IL-6 in Group II were significantly lower than Group I (*P* < 0.05). Complications, mortality, mean hospital stay, and the cost of hospitalization in Group II were 39.4 percent, 12.7 percent, 32 ± 9 days, and 30869.4 ± 12794.6 Chinese Yuan, respectively, which were significantly lower than those in Group I. The cure rate of Group II was 81.7 percent which is obviously higher than that of 59.3% in Group I (*P* < 0.05). *Conclusions*. This study indicates that the combination of PN with EN not only can improve the natural history of pancreatitis but also can reduce the incidence of complication and mortality.

## 1. Introduction

Acute pancreatitis (AP) is an acute inflammation process of the pancreas with variable involvement of other tissue or remote organ systems ranging from a mild, self-limited course requiring only brief hospitalization to a rapidly progressive, fulminant illness resulting in the multiple organ dysfunction syndromes with or without accompanying sepsis. Severe acute pancreatitis (SAP) is a common disease with emergency situation involving organ failure and/or local complications such as necrosis, abscess, or pseudocysts having mortality of up to 30 percent. Despite improvements in intensive care treatment during the past few decades, the rate of death from SAP has not significantly declined [1]. The pathogenesis of acute pancreatitis relates to inappropriate conversion of trypsinogen to trypsin and a lack of prompt elimination of active trypsin inside pancreas [2].

SAP includes a hyper catabolic state leading to protein catabolism and increased resting energy requirements [3]. As premorbid malnutrition is frequent, nutritional therapy is now recognized as an important component of SAP management [4]. The traditional approach to nutritional therapy in SAP was to rest the pancreas by way of a nil-by-mouth regimen and to deliver parenteral nutrition (PN) to meet the nutritional requirement. However, the recent studies show merits of early EN over PN [5–8]. The European Society for Clinical Nutrition and Metabolism guidelines suggest that “all patients who are not expected to be on normal nutrition within 3 days should receive PN within 24 to 48 h if EN is contraindicated or if they cannot tolerate EN” [9]. The American Society for Parenteral and Enteral Nutrition guidelines in collaboration with the Society of Critical Care Medicine state: “If early EN is not feasible or available during the first seven days following admission to the ICU, no nutrition support therapy should be provided” [10].

PN has been associated with gut mucosal atrophy, overfeeding, hyperglycemia, increased risk of infectious complications, and increased mortality rate [11, 12]. EN may be associated with high gastric residue, bacterial colonization of stomach, and increased risk of aspiration pneumonia [13]. Several studies have reported failure to deliver adequate energy intake in clinical practice [14–18], and in practice, it commonly takes up to 7 days to achieve nutritional goal by EN [19]. Nutrition in SAP has been discussed and researched over the years and still there is dominance of providing treatment which the doctors think rather than following protocols and evidence. The role of early EN is well established in SAP and should be implemented earlier. However the role of PN too cannot be ignored in SAP [20]. Since 2002, we began to use progressive combined nutritional support to cure SAP in which PN is combined to EN and we achieved good results. To discuss the mechanisms of progressive combined nutritional support in the treatment of SAP we studied retrospectively two groups of patients with two different treatments for nutritional support and compared the advantages and disadvantages of both nutritional supports.

## 2. Materials and Methods

This study is approved by the Review Board of the Second Affiliated Hospital of Chongqing Medical University, China. Total of 130 patients with severe acute pancreatitis having similar severity index, treated at The Second Affiliated Hospital of Chongqing Medical University, Chongqing, China, between January 1998 and June 2008, were retrospectively selected in to two groups, which include 58 males and 72 females with the median age of 49 ranging from 20 to 85 years old. The diagnostic criteria of severe acute pancreatitis include: clinical features, hyperamylasemia/hyperlipasemia (three times the normal upper limit); radiological evidence of severe acute pancreatitis (contrast enhanced CT scan); evidence of organ failure and/or local complications such as pancreatic necrosis; pseudocyst, abscess; computed tomography severity index (CTSI) equal to or greater than 7; Ranson score ≥3 and APACHE II score ≥8.

Group I includes 59 patients from January 1998 to December 2001 out of whom 27 were male and 32 were female whose age was between 20 to 82 years old. The median age was 51 years. More detailed characteristics of study patients are presented in Table 1. At the time of admission, the average APACHE II score was 12.21 ± 2.56 (markers of the disease at the time of admission and during the hospital stay are shown in Table 2). Group I adopted comprehensive treatment which included anti-shock therapy to maintain water, electrolyte, and acid-base; to stabilize internal environment, rational use of broad-spectrum antibiotics, sedative therapy, peritoneal lavage, organ support treatment; and treatment for the etiology of primary disease. Nutritional support was parenteral route and strictly followed by formula; Actual Energy Expenditure (AEE) = BMR × AF × IF × TF (BMR = Basic Metabolic Rate, AF = Activity Factor, IF = Injury Factor, TF = Thermal Factor).

Group II include 71 patients from January 2002 to June 2008 out of whom 31 were males and 40 were females. The patients were 20 to 85 years in age and the median age was 52.5 years. APACHE II score at the time of admission at hospital was 12.47 ± 3.71. Group II adopted progressive supportive treatment in which different course period of the disease have different nutritional supports. The first stage (up to day 3-4), the energy is calculated by formula 1/2 to 1/3 of BMR in which only glucose is administered by single parenteral route. At the second stage (from day 4 to day 7), the energy is calculated by 2/3 to 1 of BMR in which glucose accounted for 40–50 percent and fat for 50–60 percent. Both the enteral route plus parenteral route were used to achieve 100 percent target. At the third stage (day 7–10 later), the energy supplied was increased on basic requirement and strictly followed the formula AEE. At this stage the glucose accounts for 50–70 percent, fat for 30–50 percent, and the way was both the enteral and parenteral nutrition.

In this study, the nutritional therapy period is defined as the time from enrolment until the first day the patient received more than 70 per cent of their estimated nutritional requirements through volitional oral intake. PN was the provision of intravenous nutrients with the exception of ≤5% dextrose solutions. EN was defined as the provision of a nutritionally complete formula into gastrointestinal tract through a mechanical tube (gastric or small bowel tubes). EN was delivered into the jejunum distally to the ligament of Treitz. Oral intake was food taken orally by mouth. The proportion of the daily target volume of either PN or EN was calculated by dividing the delivered volume by the target volume.

At the time of hospital admission, there was no significant difference between the two groups of patients in clinical data and APACHE II score (*t*_0_ = −0.352, *P* = 0.726, *P* > 0.05).

In both the groups, we analyzed APACHE-II score, IL-6 level, serum protein level, complication rate, mortality, cure rate, length of hospital stay, and average hospital cost. Results for normally distributed outcomes are reported using medians and interquartile ranges (IQRs). A non-parametric Mann-Whitney *U*-test was used to compare the values between the two groups. All the data used SPSS 11.0 for statistical analysis. A two-sided *P* value of <0.05 was considered statically significant.

## 3. Results

### 3.1. APACHE-II Score

**Figure 1:**
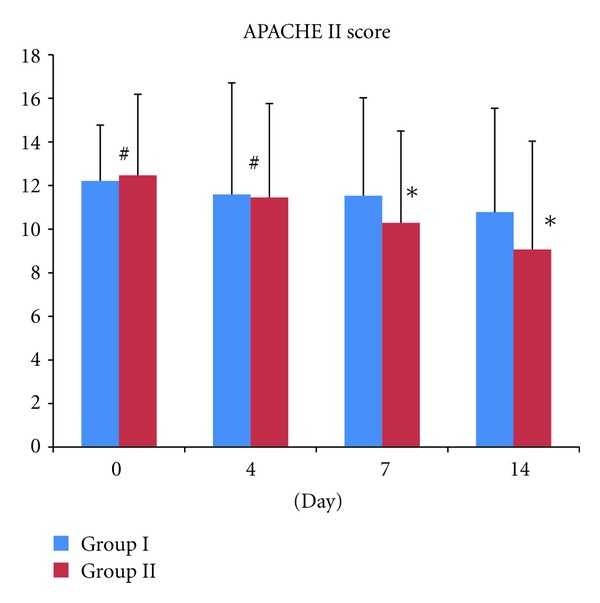
Comparison of APACHE II score on days 0, 4, 7, and 14 between Group I and Group II. ^#^ *P* > 0.05, which is not significant different between Group I and Group II on Day 1 and Day 4. However, *P < 0.05, which is statically significant between group I and Group II on Day 7 and Day 14.

In Group I, the APACHE II scores on days 4, 7, and 14 were 11.59 ± 5.12, 11.53 ± 4.49 and 10.78 ± 4.77 respectively. In Group II, on day 4, it was 11.45 ± 4.31 which shows no significant difference compared to day 4 of the Group I, *P* > 0.05. However, on day 7 and 14 after the treatment in Group II, the APACHE-II scores were 10.29 ± 4.21 and 9.07 ± 4.97, respectively, which is significantly lower than Group I, *P* < 0.05 (Figure 1).

### 3.2. IL-6 Level

**Figure 2:**
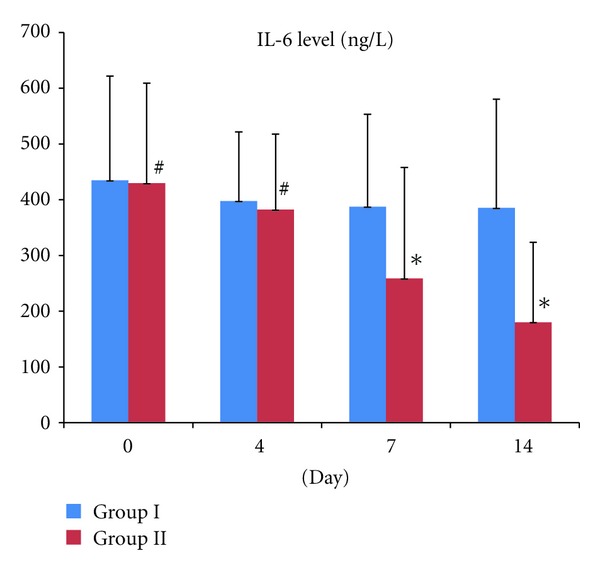
Comparison of IL-6 level on days 0, 4, 7, and 14 between Group I and Group II. ^#^ *P* > 0.05, which is not significant different between Group I and Group II on Day 1 and Day 4. However, *P < 0.05, which is statically significant between group I and Group II on Day 7 and Day 14.

Before the treatment, the IL-6 level was 434.43 ± 187.29 ng/L in Group I. After 4, 7, and 14 day of treatment, the IL-6 levels were 397.50 ± 124.15 ng/L, 387.5 ± 165.92 ng/L and 385.50 ± 194.52 ng/L, respectively. In group II, the level of IL-6 before the treatment and after 4 day of treatment was 429.57 ± 179.61 ng/L and 382.21 ± 135.73 ng/L, respectively. Comparing with Group I show no significant difference (*t*_0_ = −0.320, *P* = 0.755, *P* > 0.05; *t*_4*d*_ = −0.320, *P* = 0.755, *P* > 0.05). However, 7 and 14 day after the treatment, the IL-6 levels of Group II were 258.69 ± 199.17 ng/L and 180.33 ± 143.38 ng/L, respectively, which is significantly lower than group I (*t*_7*d*_ = 2.877, *P* = 0.016, *P* < 0.05; *t*_14*d*_ = 3.436, *P* = 0.006, *P* < 0.05) (Figure 2).

### 3.3. Serum Albumin Level

**Figure 3:**
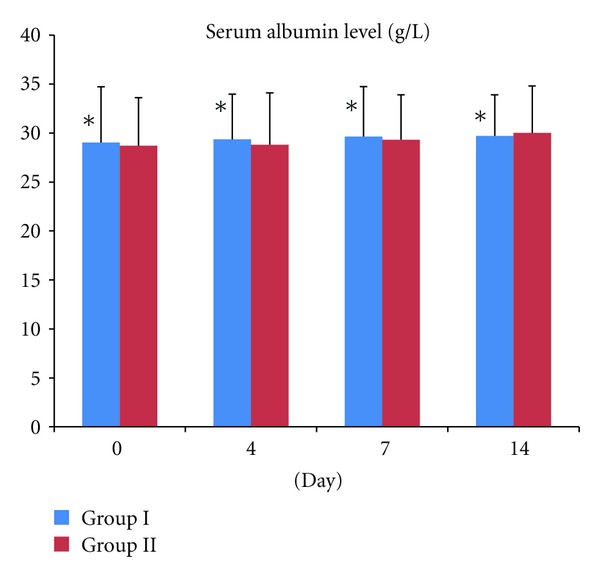
Comparison of serum albumin concentrations on days 0, 4, 7, and 14 between Group I and Group II. *P > 0.05 Group I versus Group II, which is not significant.

14 day after the treatment, the serum albumin in Group I and Group II was 29.7 ± 4.2 g/L and 30.01 ± 5.7 g/L, respectively, which is not significant between the two Groups (*t*_14*d*_ = −0.016, *P* = 0.987, *P* > 0.05) (Figure 3).

### 3.4. Complication Rate

**Figure 4:**
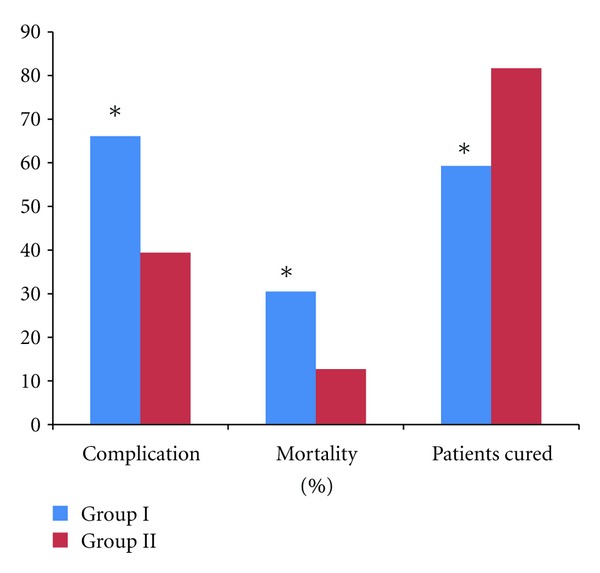
Comparison of complications, mortality, and patients-cured between Group I and Group II. *P < 0.05 Group I versus Group II, which is significant.

The complication rate of Group II is 39.4 percent which is significantly less than Group I which has 66.1 percent (*X* ^2^ = 9.173, *P* = 0.010, *P* < 0.05) (Figure 4).

### 3.5. Mortality

The mortality of Group II was 12.7 percent which is significantly less than the mortality of Group I which has mortality 30.5 percent (*X* ^2^ = 6.227, *P* = 0.044, *P* < 0.05) (Table 3).

### 3.6. Cure Rate

In Group II, the cure rate is 81.7 percent which is significantly higher than 59.3 percent in Group I (*X* ^2^ = 7.918, *P* = 0.019, *P* < 0.05) (Table 3).

### 3.7. Length of Hospitalization

The length of hospitalization in Group II was 32 ± 9 days which is significantly shorter than the 51 ± 8 days in Group I (*t* = 2.881, *P* = 0.005, *P* < 0.05) (Table 3).

### 3.8. Treatment Cost

For the Group II, it is 30869 ± 12794.6 Chinese Yuan which is significantly less than 45534.2 ± 13030.5 Chinese Yuan in Group I. (*t* = −3.475, *P* = 0.001, *P* < 0.05) (Table 3).

## 4. Discussion

Severe acute pancreatitis (SAP) is acute pancreatitis associated with complications that are either local (e.g., peripancreatic fluid collection, necrosis, abscess, pseudocyst) or systemic (e.g., organ dysfunction). According to the Atlanta Classification, SAP can be divided into two phases. The first phase of about 7–10 days start with aseptic inflammation, systemic inflammation response syndrome (SIRS), sepsis, multi organs failure (MOF), and even death [21]. The second phase usually after the second week of the disease, the circumscribed complications such as pancreatic necrosis began to appear. During this period, the lives of these patients are still in serious threat of necrotizing pancreas, complication, and death which is due to inflammatory immune response of pancreatic necrosis and infection [22]. In SAP, basal metabolic rate (BMR) increases due to inflammation and acute stress reaction thereby increase the overall energy consumption. Eighty percent of patients with severe necrotizing pancreatitis are overcatabolic and everyday lose more than 40 g of proteins which give negative balance and is adverse to the disease recovery [4, 23, 24]. Therefore, nutrition support must be guaranteed; if not in time, denutrition will get the condition worse [23]. Over the past, number of medical institutions used Harris-Benedict equation measured by resting energy expenditure (REE) of patients with SAP, but at the time when body is at stress due to the disease, there might be high metabolic decompensating state and hence exogenous nutrients may have refractoriness. Cerra et al. has proposed the concept of metabolic support which advocates, providing the necessary nutrients substrate for the body; we must also take another fact into account that it should not increase the load of the body's organs [25]. Lugli et al. has proposed the principles of the nutrition support treatment for acute pancreatitis: (a) asses the nutritional status of patients; (b) according to the severity of the disease to take the nutrition therapy; (c) confirm the patients with indications of the special nutritional support to give special way nutrition therapy [26]. All the nutritional support should supply the energy as much as possible to meet the need of body under the premise of not stimulating pancreatic exocrine function [27]. At present, very few researches about gradually combined treatment of parenteral nutrition with enteral nutrition for severe acute pancreatitis have been reported. We think this research will be interesting to the readers.

This study compares the result of two groups of SAP patients to explore the nutrition requirements of various stages of SAP and to propose the method of Gradually Combined Treatment of Nutrition Support for SAP.

In the course duration of SAP, the need for nutrients varies with the change of the duration. In order to comply with metabolism of the body in SAP, we should take the right amount of progressive nutrition support. The body's requirements for the amount of nutrients based on the balance of the body metabolic rate (BMR) and body's stress response to the inflammation of pancreatitis. At the period of stress response, the body itself is in the stage of macrophages. At this stage, the patients exhibits higher basal metabolic and catabolic rates as well as impaired metabolic capability to use exogenous amino acid and energy. As the disease goes on, the body adapts to the trauma and the tissues and organs are recovered. At this time, the body's requirements for nutritional substrate are gradually reduced and finally become close to BMR. With the stress response reduced, body's repair to trauma and anabolic enhanced, exogenous nutritional substrate requirement is gradually increased. In this stage, the energy requirement is equal to acute energy expenditure (AEE).

Till days 3-4 of the onset of SAP, a serious Systemic Inflammatory Response Syndrome (SIRS) may occur. The body is in the high catabolic stage and in stress, which represents macrophages to itself and metabolic disturbance. The principal contraindication of this phase is to improve the intracellular environment and microcirculation. Intravenous perfusion of the high calories and high viscosity nutrients solution will increase the imbalance in the intracellular environment and microcirculation. Therefore the amount of substrate required by nutritional support should be reduced, the amount should be equivalent to half of the BMR, the energy should be supplied by monosaccharide, which mainly provide to the tissue and cells rely solely, such as the brain, RBSs, and others.

After the comprehensive treatment, the maintenance of the intracellular environment and microcirculation of most patients are improved. The differences between decomposition and synthesis of metabolic in the body reduced, the phenomenon of self-macrophages gradually improved, the demand for the non-protein calories began to increase to half of BMR and requires the energy of fats as well as glucose. However, the body is still in the stress state, the intracellular environment has not fully recovered, and cells' anabolism lack vitality; high nutritional supplements will increase the burden of tissue and organ, leading to variety of metabolic complications. When SAP entered the second stage of about 7–10 days, most patients have successfully recovered through the stress period, the environment and microcirculation improved, assimilation is enhanced, demand of exogenous nutrients substrate increased. After 2 weeks, the energy demand basically reached AEE.

In this study, we analyzed APACHE-II score and IL-6 levels. After being admitted in hospital, APACHE-II score is one of the best predictor to assess the severity of pancreatitis [28]. Also the IL-6 is important parameter to show the prognostic of SAP and IL-6 > 1000 ng/L prompted a higher mortality rate [29–31]. As the time course of treatment increased, APACHE-II score and IL-6 level of both the groups were decreased. However, differences between Group I and Group II were significant on day 7 and day 14 of the admission. Also the complications, mortality, length of hospitalization, and the average cost of treatment in Group II were lower than Group I. The cure rate in Group II was higher than the Group I. On day 14 of the treatment, the serum albumin level between the two Groups was not significantly different. This is because the plasma protein levels are not a good indicator for nutrition status during inflammation due to many factors such as the acute-phase response, concomitant diseases, and the long half-life of albumin.

Patients with SAP are frequently hypercatabolic; timely institution of feeding is important if malnutrition is to be avoided or treated. Local complications of pancreatitis might cause upper gastrointestinal tract obstruction, making enteral nutrition problematic. There are also concerns that enteral nutrition may exacerbate the severity of SAP through further pancreatic stimulation and enzyme release. These considerations have led to a widespread reliance on parenteral nutrition as the main nutritional support modality in SAP. 

Many evidences suggest that there are several potential benefits to enteral nutrition compared with parenteral nutrition including a reduction in microbial translocation, improvements in gut blood flow, and preservation of gut mucosal surface immunity. Furthermore, since altered gut microbiological flora and barrier function may contribute to the development of infected pancreatic necrosis, there are theoretical advantages to enteral feeding in SAP.

About the timing of nutritional support for the patients with SAP, in most of the studies, both parenteral nutrition and enteral nutrition begin within 48 h; parenteral nutrition is started later than enteral nutrition, more likely an assistant method of enteral nutrition [32]. Although enteral nutrition is a more beneficial nutrition support, it is not easy to implement at early time and has high risk [33]. In Group II, parenteral nutrition was used at the first stage in order to avoid excessive irritation in severe stress period. In the second and third stages, parenteral and enteral nutrition were used together to make up for each other's deficiencies.

## 5. Conclusion

In severe acute pancreatitis, evaluation of body's metabolism should be the first consideration and then gradually combined treatment of parenteral nutrition with enteral nutrition should be used as routine therapy. This cannot only improve the natural history of pancreatitis but also can reduce the incidence of complication and mortality.

## Figures and Tables

**Table 1:** Characteristics of study patients.

	Group I	Group II	*P* value
	*n* = 59	*n* = 71	
Age in years (average)	51	52.5	
Male	27	31	
Female	32	40	
Etiology			
Gallstones	25 (49.01%)	34 (47.88%)	
Alcohol	21 (41.17%)	28 (39.43%)	
Idiopathic	3 (5.88%)	6 (8.45%)	
Drug Induced	2 (3.92%)	3 (4.22%)	
Duration of symptom of disease at the time of admission (days in mean ± SD and range)	2.63 ± 0.73 (1–5)	2.77 ± 1.01 (1–5)	0.247^a^

^a^Mann-Whitney *U*-Test.

**Table 2:** Markers of disease at the time of admission and during the hospital stay.

	Group I	Group II	*P* value
	Mean ± SD (range)	Mean ± SD (range)	Group I versus Group II^a^
APACHE-II Score			
Day 0	12.21 ± 2.57	12.47 ± 3.71	0.363
Day 4	11.59 ± 5.12	11.45 ± 4.31	0.276
Day 7	11.53 ± 4.49	10.29 ± 4.21	0.010
Day 14	10.78 ± 4.77	09.07 ± 4.97	0.009
IL-6			
Day 0	434.43 ± 187.29 ng/L	429.57 ± 179.61 ng/L	0.755
Day 4	397.50 ± 124.15 ng/L	382.21 ± 135.73 ng/L	0.716
Day 7	387.50 ± 165.92 ng/L	285.69 ± 199.17 ng/L	0.016
Day 14	385.50 ± 194.52 ng/L	180.33 ± 143.38 ng/L	0.006
Serum Albumin Level			
Day 0	28.6 ± 3.7 g/L	30.03 ± 6.2 g/L	0.963
Day 4	29.36 ± 4.6 g/L	28.8 ± 5.3 g/L	0.865
Day 7	29.64 ± 5.1 g/L	29.3 ± 4.6 g/L	0.872
Day 14	29.7 ± 4.2 g/L	30.01 ± 5.7 g/L	0.987

^a^Mann-Whitney *U*-test.

**Table 3:** Outcome in the two groups.

	Group I	Group II	*P* value
Complication rate	66.1%	39.4%	0.010^a^
Mortality	30.5%	12.7%	0.044^a^
Cure rate	59.3%	81.7%	0.019^a^
Length of hospitalization	51 ± 8 days	32 ± 9 days	0.005^b^
Treatment cost	¥ 45534 ± 3031.5	¥ 30869 ± 12794.6	0.001^b^

^a^Mann-Whitney *U*-test.

^b^Fisher's exact test.
